# Endothelin and the tumor microenvironment: a finger in every pie

**DOI:** 10.1042/CS20240426

**Published:** 2024-05-24

**Authors:** Philipp F. Arndt, Kati Turkowski, Michael J. Cekay, Bastian Eul, Friedrich Grimminger, Rajkumar Savai

**Affiliations:** 1 Lung Microenvironmental Niche in Cancerogenesis, Institute for Lung Health (ILH), Justus Liebig University, Giessen, Germany; 2 Department of Internal Medicine, Justus-Liebig-University Giessen, Universities of Giessen and Marburg Lung Centre (UGMLC), Member of the Cardio-Pulmonary Institute (CPI), Member of the German Centre for Lung Research (DZL), Giessen, Germany; 3 Max Planck Institute for Heart and Lung Research, Member of the DZL, Member of the CPI, Bad Nauheim, Germany

**Keywords:** Cancer, endothelin, epithelial to mesenchymal transition, hypoxia, tumor microenvironment

## Abstract

The tumor microenvironment (TME) plays a central role in the development of cancer. Within this complex milieu, the endothelin (ET) system plays a key role by triggering epithelial-to-mesenchymal transition, causing degradation of the extracellular matrix and modulating hypoxia response, cell proliferation, composition, and activation. These multiple effects of the ET system on cancer progression have prompted numerous preclinical studies targeting the ET system with promising results, leading to considerable optimism for subsequent clinical trials. However, these clinical trials have not lived up to the high expectations; in fact, the clinical trials have failed to demonstrate any substantiated benefit of targeting the ET system in cancer patients. This review discusses the major and recent advances of the ET system with respect to TME and comments on past and ongoing clinical trials of the ET system.

## Introduction

**Figure 1:**
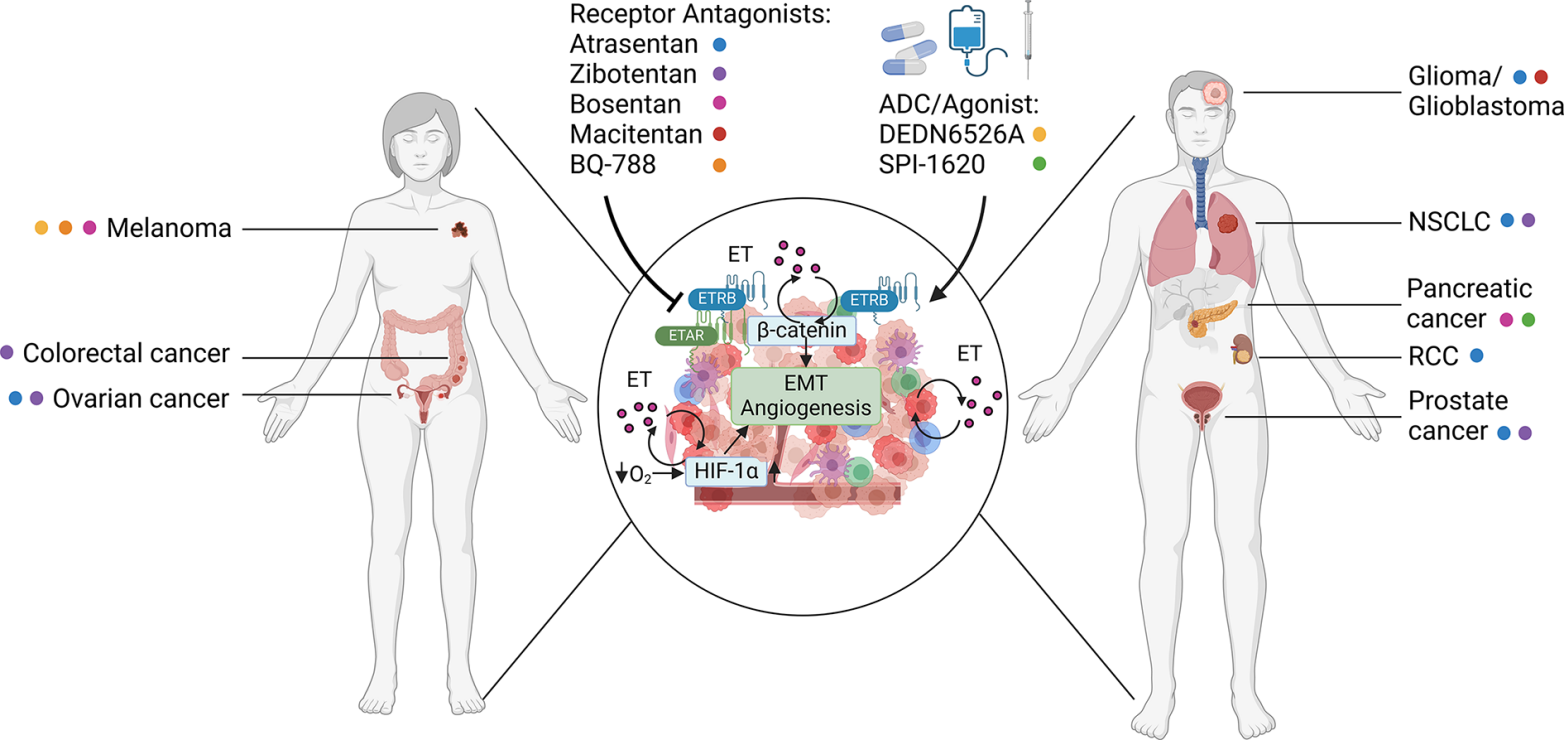
The ET system influences the TME the TME in various cancer types. Within the TME the hypoxia/HIF-1α axis, the β-catenin axis, and its ability to activate immune cells that in turn secrete ET, the ET system is at the center of several autocrine, self-sustaining circuits that promote angiogenesis and EMT. This significant influence of the ET system on cancer progression has prompted numerous preclinical and clinical studies in various cancers such as skin, lung, ovarian and prostate cancer, in which different agents have been used to disrupt the ET system (Created with BioRender.com).

Although endothelins (ETs) are known to be the most potent vasoconstrictors in the human cardiovascular system [1], abnormal activation of the ET system has been recognized as a driving force behind cancer progression in various human tumor types such as prostate, ovarian, skin, breast, colon, and lung cancers, as well as leukemia [2–9]. This abnormal activation can be promoted by ET overexpression, dysregulation of receptor expression or loss of a negative regulator. The ET system is embedded in an extensive and complex signaling network and interacts with numerous other cellular pathways [10]. The cell population of the tumor microenvironment (TME) is heterogeneous and varies depending on the type of tumor, but characteristic features are immune cells, stromal cells and their interaction with cancer cells [11–13]. The central role of the TME in cancer progression is highlighted by the success of immune checkpoint inhibitors (ICI), which target the interaction between tumor cells and T cells in a growing number of cancer types [14]. The TME provides a spatial and temporal context in which the activation of the ET system has tumor-promoting effects. Thus, the ET system can induce proliferation, inhibit apoptosis, stimulate angiogenesis, promote invasion and metastasis and even modulate the TME [10]. Early studies have consistently shown that ET1 has pro-proliferative and anti-apoptotic properties. In these studies, endothelin A receptor (ETAR) antagonists were found to reduce proliferation and restore apoptosis in various cancer cell lines, including prostate, cervical and ovarian cancers and leukemia cells [15–19]. This pro-tumor effect is thought to be mediated via the extensive interaction with other well-established signaling pathways such as mitogen-activated protein kinase (MAPK), AKT, nuclear factor ‘kappa-light-chain-enhancer’ of activated B-cells (NF-κB), β-catenin and RHO [8,10], which promote cell proliferation and synergistically impair cell apoptosis. In a recent study, the endothelin system was shown to promote colorectal cancer progression via signal transducer and activator of transcription (STAT)3 phosphorylation [20]. However, in addition to its direct effect on the proliferation and survival of cancer cells, the ET system also has a profound effect on the TME. As the ET system modulates the hypoxia/hypoxia inducible factor (HIF)-1α and the β-catenin axes and activates immune cells that subsequently release ET, the ET system is central to many self-sustaining circuits within the TME, inducing angiogenesis and promoting epithelial-to-mesenchymal transition (EMT). The profound impact of the ET system on the TME justified a vast amount of preclinical and clinical investigations across diverse cancer types, including skin, lung, ovarian, and prostate cancer. These studies have explored various agents including atrasentan, zibotentan, bosentan, macitentan, and BQ788 aimed at disrupting the ET system in an effort to modulate the TME and impede cancer advancement (Figure 1). This review aims to highlight the crucial role of the ET system in carcinogenesis, with a focus on the TME. In addition, it summarizes and comments on recent and ongoing clinical trials targeting the endothelin system.

## The endothelin system

**Figure 2:**
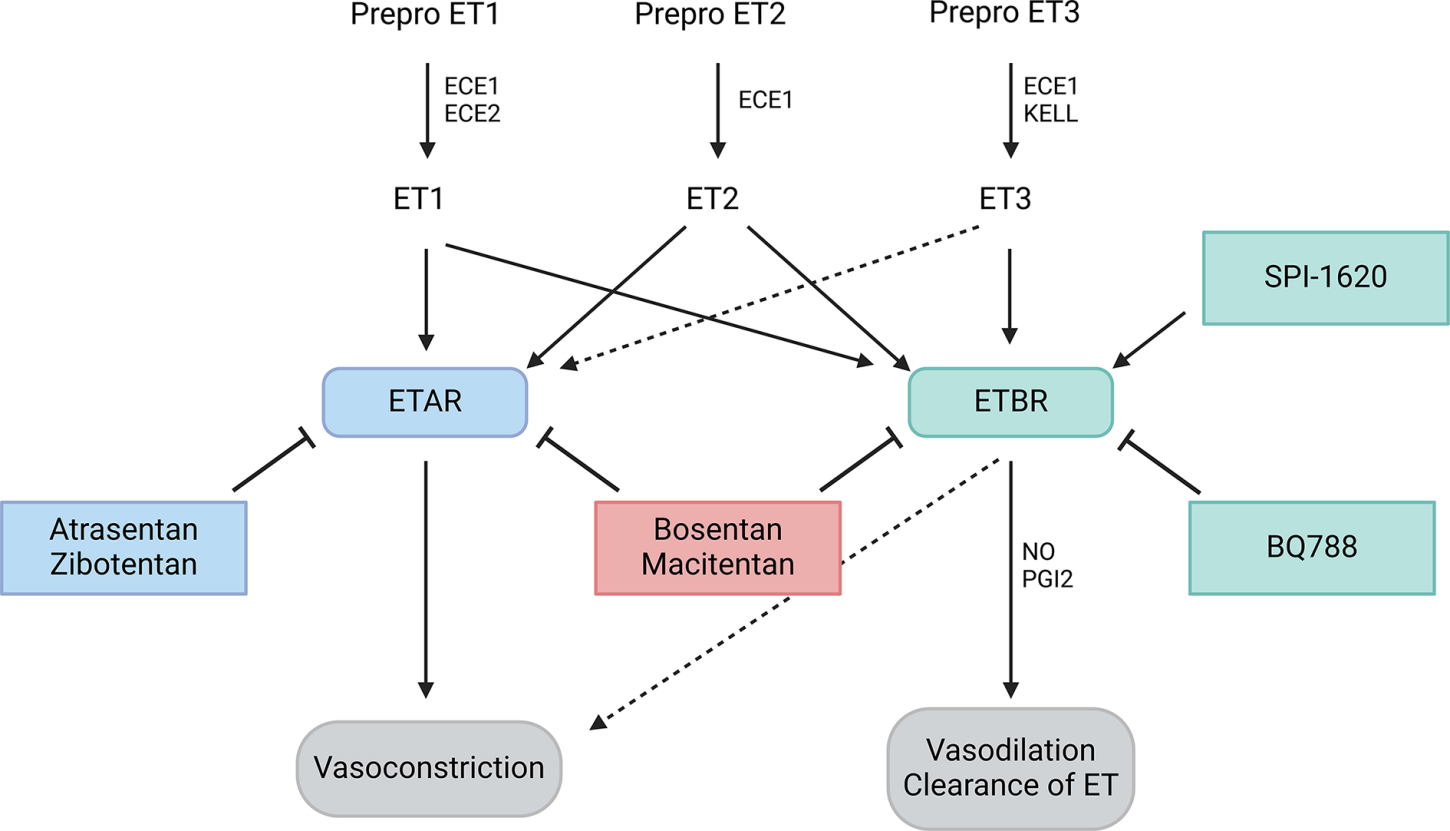
The ET system. The ETs are derived from prepro ET and are converted into their active form by ECE. ET1 and ET2 bind to the ETAR and the ETBR with equal affinity. However, ET3, the less highly expressed form of ET, has a higher affinity for ETBR. ETAR then causes smooth muscle cell contraction via activation of G-proteins, phospholipase C (PLC) and inositol 1,4,5-triphosphate (IP3) to mediate vasoconstriction. Activation of ETBR causes vasodilation via the release of NO and prostaglandin I2 (PGI2). Under certain conditions, ETBR can mediate vasoconstriction, but not in a clinically relevant manner. Atrasentan and zibotentan are specific inhibitors of ETAR, while BQ788 is a specific antagonist of ETBR. Bosentan and macitentan inhibit both ETAR and ETBR. SPI-1620 is an ETBR agonist (Created with BioRender.com).

The ETs consist of three 21 amino acid peptides ET1, ET2, and ET3, which are derived from the three genes endothelin (EDN)1, EDN2, and EDN3. Regulation of ET gene expression occurs primarily at the level of transcription [21], although epigenetic mechanisms such as DNA methylation [22] or histone modifications [23] and microRNA (miRNA) interference [24–26] have been shown to influence EDN1 mRNA levels. Important transcription factors such as forkhead box O (FOXO) 1 and HIF-1α are among the known regulators of ET1 expression [21]. These genes encode the precursor molecules pre-pro-ET, which are first cleaved by an endopeptidase and then again by the endothelin-converting enzyme (ECE) to form the highly vasoactive ETs. This vasoactive effect is mediated by binding to two G-protein-coupled receptors, the ETAR, which leads to vasoconstriction, and the endothelin B receptor (ETBR), which causes vasodilation through the production of nitric oxide (NO) (Figure 2). ETAR is predominantly expressed in vascular smooth muscle cells, fibroblasts, and cardiomyocytes, while ETBR is mainly found on vascular endothelial cells (ECs) [27]. In the context of cancer, ETAR is strongly expressed in cancer cells, while ETBR is more strongly expressed in other cells of the TME [10]. ET1 and ET2 bind to ETAR and ETBR with the same affinity, but ET3 shows a higher affinity to ETBR than to ETAR [28]. In addition to their vasoactive effect, binding to the receptors activates a cascade of other signaling pathways, G-protein-independent and often via the central scaffold protein β-arrestin1 [29,30]. These signaling pathways include NF-κB, epidermal growth factor receptor (EGFR), vascular epithelial growth factor receptor (VEGFR), and β-catenin, signaling pathways known for their central role in carcinogenesis [8,31–33]. There are two known mechanisms for ET degradation, one via ETBR by internalization and lysosomal degradation and the other via extracellular neutral endopeptidase (NEP) by direct cleavage [28]. The peptides, the genes, the cleavage enzymes and the receptors together form the ET system, each of which represents a target for possible therapeutic intervention. Most clinical trials employed an ET receptor antagonist such as atrasentan and zibotentan, specific inhibitors of ETAR, or BQ788, a specific antagonist of ETBR. Bosentan and macitentan are dual receptor antagonists, exerting their effects by inhibiting both ETAR and ETBR. Fewer clinical trials investigated other modes of intervention such as SPI-1620, an agonist for ETBR (Figure 2).

## The endothelin system and the tumor microenvironment

Despite progress in cancer treatment, it remains a leading global cause of death, challenging researchers to find new therapies. Tumors, comprising of not only cancer cells but also stromal, immune, and ECs, create a diverse microenvironment (TME). This complex mix influences cellular architecture, hypoxia, metabolite availability, extracellular matrix (ECM) aberrations and EMT, collectively shaping the environment [12,34–36]. The TME is not merely an appendage to tumor cells; it can either promote or hinder cancer progression based on its cellular composition [37,38]. The endothelin system plays a crucial role in shaping TME features by regulating responses to hypoxia, ECM degradation, EMT and cellular composition, as explained in the following sections.

## Hypoxia, EMT, and ECM degradation

**Figure 3:**
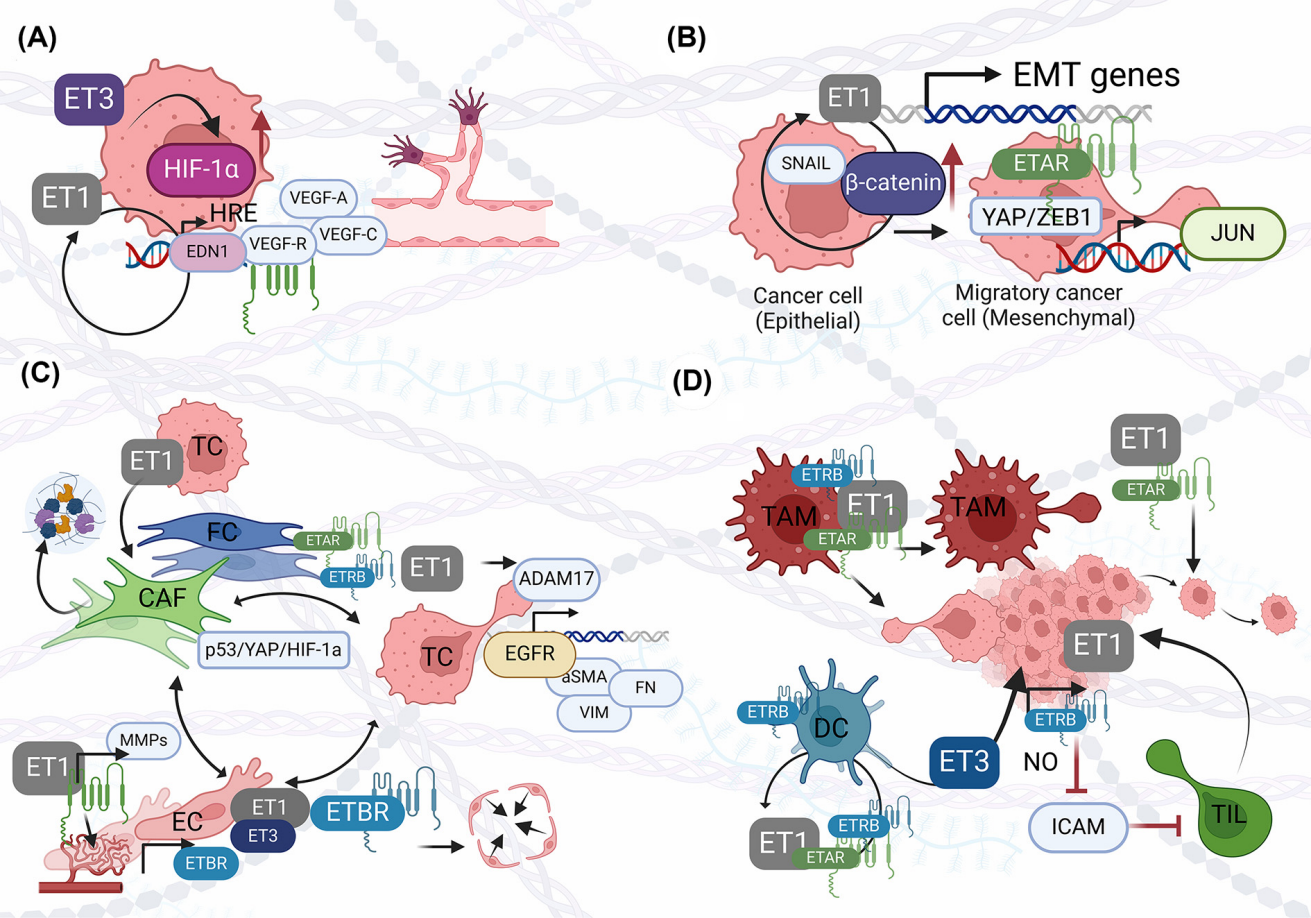
The ET system in TME. (**A**) ET1 and ET3 increase HIF-1α levels and activate the transcription of angiogenesis genes, leading to increased neovascularization. HIF-1α itself promotes transcription of EDN1, creating a feedback loop that further drives angiogenesis and tumor cell aggressiveness. (**B**) ET1 up-regulates SNAIL and promotes β-catenin activity to promote EMT. END1 as a transcriptional target of β-catenin, creating a self-sustaining loop. ETAR orchestrates nuclear translocation of the YAP/ZEB1 complex as a co-activator of downstream pro-mesenchymal gene expression of JUN. (**C**) ET1 can support fibrocyte (FC) and CAF behavior by inducing proliferation, migration, contraction and ECM protein production. ET1 causes increased motility via activation of EGFR signaling mediated by ADAM17, as well as induction of α-SMA, vimentin, and fibronectin. The ET axis mediates cross-talk between fibroblasts, ECs and cancer cells via the p53/YAP/HIF-1α complex, which increases migration and aggressiveness. Binding of ET1 to ETBR promotes EC proliferation, migration and tube formation via up-regulation of MMPs. In addition, ET1 mediates the contraction of ECs via ETBR to control permeability. (**D**) TAMs express both ET receptors. ET-1 activates TAMs to secrete pro-inflammatory cytokines and ET-1; in addition, macrophages are known to migrate toward ET-1 and promote cancer cell migration, which enables metastasis. DCs express both ET receptors, with ETAR promoting maturation, survival, IL-12 secretion and stimulation of T cells, forming a self-regulatory loop. ETBR mediates DC apoptosis. ET-3 induces DCs to infiltrate the TME and promote tumor growth. ETBR inhibits the endothelial ICAM1 via the release of NO, thus preventing T-cell homing, which promotes immune response and tumor growth (Created with BioRender.com).

Hypoxia is a characteristic feature of the TME as it controls angiogenesis and triggers EMT via HIF-1α [39]. Several studies have investigated the cellular response to a hypoxic microenvironment and revealed a central role of the endothelin axis, especially in its interaction with HIF-1α. Under normoxic conditions, HIF-1α is hydroxylated by the prolyl hydroxylase domain (PHD) 2, leading to its degradation by the cell’s own proteasome [40]. Under hypoxic conditions, the absence of this hydroxylation causes HIF-1α to accumulate and translocate to the nucleus, where it binds to hypoxia response element (HRE) binding sites to activate transcription of VEGFA, VEGFC and VEGFR, triggering angiogenesis and lymphangiogenesis in response to hypoxia [41]. Treatment of tumor cells with ET1 and ET3 resulted in increased HIF-1α levels, increased aggressiveness and neovascularization (Figure 3A).

In addition, silencing ET1 or using ETR inhibitors has been shown to lower HIF-1α and VEGF levels in endothelial [42] cells and reduce angiogenesis in chondrosarcoma [43], ovarian cancer [44] and melanoma cells [33,45]. The endothelin axis modulates the TME via HIF-1α in two known cases: ET1 has been shown to stabilize HIF-1α by inhibiting PHD2 in melanoma [46] and lymphoid ECs, mimicking [47] and enhancing hypoxia in the TME. Second, ETAR activation couples the scaffold protein β-arrestin to HIF-1α, which was critical for transcription of HIF-1α target genes in ovarian cancer cells [48]. Furthermore, HIF-1α itself promotes transcription of EDN1 [21], creating a feedback loop in which ET1 plays a central role and significantly modulates the TME. Recent research has shown that the combination of metformin and simvastatin inhibits tumor progression and attenuates hypoxia. Reduced expression of EDN1 in RNA sequencing and reduced HIF-1α levels point to the ET axis as the driving mechanism, but further studies are needed to conclusively demonstrate the causality of this relationship [49]. HIF-1α in a complex with yes-associated protein (YAP), mutant p53 and β-arrestin was also recently identified as an escape mechanism of high-grade serous ovarian carcinoma (HG-SOC) cells for poly-ADP-ribose polymerase (PARP) inhibition. Application of the dual ET receptor antagonist macitentan was able to restore sensitivity to PARP inhibition in HG-SOC xenograft mice [50]. In addition, ET1 promotes cyclooxygenase (COX)1 gene expression and increases prostaglandin E2 (PGE2) production under hypoxic conditions, which in turn leads to an increase in VEGF and matrix metalloproteinase (MMP) and promotes tumor angiogenesis and invasiveness [51–53].

EMT is critical for development, but also contributes to cancer progression and is a hallmark of TME. EMT is a process in which the epithelial and mesenchymal properties of a cell are down-regulated and up-regulated. This process is accompanied by the breakdown of epithelial cell–cell junctions and the polarity of the cell, giving the cell the ability to migrate. Together with the degradation of the ECM, this allows the cells to invade and metastasize, increasing the aggressiveness of the cancer [54]. Activation of the ET axis can trigger EMT through multiple mechanisms (Figure 3B), including up-regulation of the EMT-associated transcription factor SNAIL at transcriptional and posttranslational levels [55,56] and promotion of β-catenin activity, which allows nuclear translocation to act as a transcription factor to promote a pro-mesenchymal and pro-tumor phenotype. Mechanistically, ET1 prevents the degradation of β-catenin by inhibiting glycogen synthase kinase (GSK) 3β, it also promotes its phosphorylation via the Src pathway and finally its activation of β-arrestin enables its nuclear translocation [10]. Interestingly, EDN1 is a transcriptional target of β-catenin [57], creating a second self-sustaining loop similar to the HIF-1α/ET1 axis. This emphasizes the integral role of ET1 in shaping the TME. ET1 treatment of ovarian cancer cells leads to the typical features of EMT, such as loss of expression of adherens and tight junction proteins, E-cadherin and β-catenin, and increase in expression of N-cadherin and vimentin [58]. In addition, ET receptor blockade was shown to overcome therapy resistance of ovarian cancer and colorectal cancer cells, which was associated with decreased expression of EMT markers [59,60]. Therapy resistance of ovarian cancer cells was also overcome by miR-30a by directly targeting the 3’UTR of ETAR, which was associated with a decrease in EMT markers (Figure 3B) [61]. In further studies on microRNAs, the ET axis was shown to induce EMT by targeting miR-300 and miR-489, both of which inhibit TWIST in chondrosarcoma and oral mucosal cancer cells [62,63]. In a more recent study, ETAR was shown to orchestrate the nuclear translocation of the YAP/Zinc finger E-box-binding homeobox1 (ZEB1) complex, which acts as a co-activator of JUN to activate pro-mesenchymal transcription. The up-regulation of this specific gene signature in ovarian cancer appears to correlate with a poorer prognosis of patients [64]. Furthermore, ET1 has been shown to interfere with cell–matrix and cell–cell communication by modulating the expression of various integrins and inducing phosphorylation of connexin 43 via the Src pathway in ovarian cancer and melanoma cells [65–67].

ET1 is known to regulate the MMP family and the urokinase plasminogen activator (uPA) system. ET1 has been shown not only to increase the expression of various MMPs but also to reduce the expression of TIMP 1 and 2 (TIMP = tissue inhibitors of MMP1), leading to rapid ECM degradation and subsequently to increased metastasis and invasion in ovarian cancer and Kaposi's sarcoma [68,69]. Several studies have shown that ET1 increases the activation and expression of MMP2, -9 and -13 in colon cancer, chondrosarcoma, osteosarcoma, and glioblastoma cells via focal adhesion kinase (FAK), phosphatidylinositol 3-kinase (PI3K), AKT, mammalian target of rapamycin (mTOR), and NF-κB [69–72]. Recent studies have found that IQ-domain GTPase-activating protein 1 (IQGAP1), integrin-linked kinase (ILK), and the scaffold protein β-arrestin are required for ETAR-mediated MMP production, invadopodia formation, migration, and invasion in ovarian cancer cells [73,74].

Induction of typical EMT features, degradation of the ECM and emulation of hypoxia are the main mechanisms by which the ET system modulates the TME and acts as a pro-tumor agent. In particular, the self-sustaining loops in the ET-HIF-1α and ET-β–catenin axis emphasize the central role of the ET system in the TME and consequently in cancer progression.

## Fibrocytes and stromal cells within the TME

It has been shown that stromal cells within the TME promote tumor progression in various cancers through involvement of the ET system (Figure 3C) [50,75]. Recently, it has been demonstrated that fibrocytes have a prominent role in boosting the lung cancer-supporting niche, extending far beyond their role in wound healing or tissue remodeling processes and the endothelin system is a major molecular player in fibrocyte-driven cancer supporting niche organization [75]. Also, it was shown that the ET system can be induced by stromal cells such as fibrocytes and cancer-associated fibroblasts (CAFs) in different cancer types [75–77]. Moreover, tumor cells and fibrocytes itself showed an increase in the mRNA expression for endothelin receptor A and B and ET1 in lung cancer cell lines upon co-culture. Furthermore, triple co-cultures including fibrocytes, cancer cells and macrophages not only enhanced pro-proliferative and pro-migratory markers of cancer cells, shifted the macrophages toward a M2-like phenotype and increased phenotypic maturation markers in fibrocytes, but also an up-regulation of both ETA and ETB was detected in the separated cell fractions correlating with increased expression of both endothelins in human and mouse lung cancer samples [75]. CAFs in contrast are a heterogeneous cell population composed of multiple cells of origin and, as one of the key players in tumorigenesis [78], can support the invasiveness of oral cancer cells through paracrine signaling upon ET1 treatment [79]. In addition, ET1 released from tumor cells can also affect the behavior of isolated CAFs from tumor and adjacent tissues by inducing proliferation, migration, contraction, and the production of ECM proteins from various cancers [10]. Further, it was shown that, ET1 treatment of fibroblasts cause increased motility of head and neck cancer cells via activation of EGFR signaling mediated by ADAM17 [50,79,80], as well as induction of activated fibroblast markers such as α-SMA (smooth muscle actin), vimentin and fibronectin [80], indicating a contribution to ECM remodeling. In addition, the ET axis mediates an interaction between fibroblasts, ECs and ovarian cancer cells via the p53/YAP/HIF-1α complex, which increases EC and fibroblast migration as well as cancer cell migration and aggressiveness [50]. Furthermore, stromal cells and the endothelin axis are associated with tumor progression, as shown in a mouse model of lung cancer in which fibrocytes within the TME are required for the antitumor effect of the known ET receptor antagonist bosentan as shown by suppression of migration and sprouting mediated by the secreted cytokines within the TME [75]. In addition, ET antagonism plays an important role in inhibiting the tumor-promoting properties of fibroblasts depending on tumor type, as shown in colorectal cancer, where selective antagonism of ETAR prevents ET1-stimulated proliferation of fibroblasts, while antagonism of ETBR inhibits ET1-mediated contraction [81]. Inhibition of oral cancer cell invasion stimulated by ET1-treated fibroblast-conditioned media was blocked by both ETAR and ETBR antagonists without selectivity [79].

## Endothelial cells and the endothelin system

ECs form the lining of a vessel and facilitate vasodilation through the release of NO mediated by ETBR. In addition, ECs are an integral part of the TME and actively participate in the cellular cross-talk that drives cancer progression. It is known that ECs not only express ETBR, but also produce ET1 and ET3. It has been shown that hypoxia, one of the main features of TME, stimulates ET1 production in ECs [45]. In addition, binding of ET1 to ETBR promotes proliferation, migration and tube formation of human umbilical vascular ECs (HUVECs) [82] and lymphatic ECs [42]. Furthermore, invasiveness was increased as both were shown to increase MMP production upon ET1 stimulation [83], emphasizing the important role of ET and ECs in cancer progression and angiogenesis. In addition, ECs are involved in the cellular crosstalk that forms the TME by communicating with macrophages, T cells and other stromal cells via the ET system (Figure 3C), which is discussed in the relevant sections of the review [50,84,85]. In another study, brain ECs were shown to mediate chemoresistance of breast and lung cancer cell lines via ET – integrin signaling, which could be abrogated by dual ET receptor antagonism [86]. In a translational study, ET1 was measured in the serum of patients with castration-resistant prostate cancer before and 2–5 weeks after treatment with docetaxel, a standard chemotherapeutic agent, to determine its prognostic value for survival. ET1 levels were not predictive of patient survival, but a 3.8-fold or higher increase in circulating ECs 2–5 weeks after treatment was found to predict worse survival (10.9 months versus 16.8 months) [87]. This particular finding could contribute to biomarker profiling but should be evaluated in a prospective setting.

## Immune cells within the TME

ET polarizes and promotes the migration and differentiation of immune cells that invade the tumor and thus significantly influences the TME and subsequently the progression of cancer.

### Macrophages

TAMs produce ET and express both ET receptors and are known to migrate toward ET, mediated by both ETAR and ETBR [88,89]. Macrophages and THP1 cells in particular migrate towards ET, whereas undifferentiated monocytes do not [88]. In another study, ET1 was shown to promote migration and invasion of both bladder cancer cells and macrophages via ETAR (Figure 3D). Furthermore, depletion of ET1 and pharmacologic antagonism of ETAR prevented lung metastasis in a xenograft mouse model. In the same study, macrophages were polarized after ET1 stimulation, leading to the production of various proinflammatory cytokines such as IL-6, COX2, and MMPs [89]. In addition, macrophage infiltration and mammary cancer tumor growth were inhibited in ETBR-deficient mice [90]. Thus, while ET activates macrophages, cytokines produced by these macrophages were shown to increase ET production and receptor expression on HUVECs and breast cancer cells, facilitating the migration of breast cancer cells to ECs [84]. This study underscores the importance of macrophages and ET in breast cancer metastasis and demonstrates another paracrine self-sustaining circuit within the ET system. Further interesting results show a positive correlation between EDNRA expression, poorer overall survival (OS) and macrophage infiltration in gastric cancer. EDNRA was significantly associated with M2-like pro-tumoral macrophage polarization, further highlighting the importance of ET in shaping the TME [91].

### Dendritic cells

Dendritic cells (DCs) are a group of highly specialized antigen-presenting cells that are known to infiltrate the tumor stroma. As such, these cells play a central role in the antigen-specific immune response, which is why their activation potentially triggers very effective anti-tumor immunity [92]. DCs are known to produce ET and express ET receptors, which increases during maturation. Interestingly, ETAR and ETBR have opposite effects on DC activation. While ETAR blockade inhibited DC maturation, survival, IL-12 secretion and T cell stimulation, ETBR blockade resulted in increased DC maturation and decreased DC apoptosis [93]. Therefore, an autocrine circuit appears to regulate DC maturation and function. However, it remains unclear which receptor is dominant in DC in the context of the TME. This question deserves further investigation, as manipulation of DCs via the ET system may represent a mechanism to overcome ICI therapy resistance. In a recent study, ET3-overexpressing mice exhibited larger tumors and higher levels of DCs and T-regulatory cell infiltrates (Figure 3D). ETRB blockade itself prevented tumor growth; it also significantly increased sensitivity to ICI in a melanoma mouse model [94].

### Lymphocytes

The complex regulatory interplay between ET and lymphocytes within the TME is still a poorly understood phenomenon. However, an early study in ovarian cancer showed that overexpression of ETBR was closely associated with the absence of tumor-infiltrating lymphocytes (TIL) and worse OS. Furthermore, adhesion of T cells to the endothelium was inhibited by ET1, which occurs through ETBR-mediated NO release and suppression of the endothelial intercellular adhesion molecule (ICAM1). ETBR antagonism restored homing and adhesion of T cells to the endothelium and supported immunotherapy in a mouse model of ovarian cancer [85]. Similar results have been documented in studies of central nervous system malignancies in which patterns of endothelin receptor expression were examined in primary central nervous system lymphomas, glioma samples, and glioblastoma cell lines. These studies revealed that the expression of ETBR by lymphoma and ECs may play an essential role in mediating the trafficking of TILs (Figure 3D). In addition, samples with an ETBR expression level of 50% or more had a lower number of infiltrating cytotoxic T lymphocytes and a higher number of infiltrating T regulatory lymphocytes than samples with an ETBR expression level of less than 50%. These results suggest that ETBR expression during neoangiogenesis may impede the colonization of cytotoxic T cells around the tumor and thus contribute to immune escape mechanisms in gliomas [95,96]. In line with this, another study found that the expression of ETRB is closely associated with a higher clinical stage of glioma and thus a poorer prognosis [97]. In another study by Son et al., polymeric nanoparticles containing macitentan, a dual endothelin receptor antagonist, were found to successfully regulate cancer-associated fibroblast function and increase cytotoxic T-cell colonization, while significantly reducing T-regulatory lymphocytes in an orthotopic mouse model of breast cancer. Interestingly, polymeric nanoparticles with macitentan were not able to significantly reduce tumor size. However, when combined with a programmed death (PD)-1 antibody, tumor growth was inhibited by 90.1% [98]. In a study on squamous cell carcinoma of the esophagus, ETBR status was an independent prognostic factor for poorer OS. In contrast with the previously mentioned studies, no significant correlations between TILs and ETBR expression were observed [99]. In another recent study, macitentan enhanced cytotoxic T cell-mediated tumor killing by reducing the binding of PD-1 to extracellular vesicles carrying programmed death ligand (PDL)-1. In addition, the combination of macitentan and an anti-PDL-1 antibody significantly improved anti-tumor efficacy by increasing the homing of cytotoxic T cells and reducing the number of T-regulatory lymphocytes. Interestingly, ETBR was not involved in the mechanism of action in this study, but ETAR [100]. In summary, the ET system prevents the colonization of T cells in the TME and thus represents a mechanism of immune defense. The use of ET receptor antagonists in combination with ICIs significantly reduced tumor growth in various preclinical cancer models and thus represents a viable option for clinical trials. The ET axis influences the TME in several ways. It is at the center of several autocrine, self-sustaining loops that amplify the effects of hypoxia, drive EMT, and promote the polarization of pro-tumoral macrophages. Furthermore, ET inhibits the colonization of TIL in the TME and thus promotes cancer progression. Several different animal models have shown significant efficacy of ETR antagonists, especially in combination with ICI.

## Clinical trials interfering with the endothelin system

There are many reasons for targeting the endothelin system for the treatment of cancer in clinical trials. Aside from the promising preclinical data mentioned earlier, many solid tumor types express ETAR and/or ETBR, and overexpression of the receptors is associated with poorer patient survival or metastasis [10,91,101,102]. However, none of the clinical trials targeting the endothelin axis have shown positive results in cancer patients.

## ETAR antagonists

Since ETAR is the receptor that is overexpressed in most cancers [10], clinical trials have been conducted with various ETAR antagonists such as atrasentan and zibotentan in various solid cancers such as glioma, metastatic and non-metastatic prostate cancer, lung, ovarian and renal cell cancer as monotherapy or in combination with chemotherapy [103–109] **(**Table 1).

**Table 1:** Cancer types evaluated in past clinical trials targeting the endothelin system

Cancer type	Compound	Targeting	NCT number	Trial design	References
**NSCLC**	Atrasentan	ETAR	None	Phase I/II, single-arm, plus carboplatin and paclitaxel	[106,110]
	Zibotentan		NCT00745875	Phase II, placebo-controlled, plus pemetrexed	
**Melanoma**	BQ-788	ETBR	NCT02442466	Proof of concept, intralesional injections	[111–113]
	Bosentan	ETAR and ETBR	NCT01009177	Phase II, multicentre, open-label, single-arm, monotherapy	
	DEDN6526A	ETBR ADC	NCT01522664	Multicentre, double-blind, placebo-controlled, randomized, plus dacarbazine	
				Phase I, open-label, single arm	
**Kidney**	Atrasentan	ETAR	NCT00039429	Phase I, single-arm, plus IFN-α subcutaneous injection	[108,114]
			NCT00039429	Phase II, single-arm, monotherapy	
**Ovarian**	Atrasentan	ETAR	NCT00653328	Phase II, multicentre, single arm, plus PLD	[109,115]
	Zibotentan		NCT00929162	Phase II, randomized, double-blind, placebo-controlled, plus carboplatin and paclitaxel	
**Prostate**	Atrasentan	ETAR	NCT00036556	Phase III, randomized, monotherapy	[107,109,116–118]
	Zibotentan		NCT00038662	Phase II, randomized, double-blind, placebo-controlled, monotherapy	
	YM598		NCT00046943	Phase III, open-label, multicentre, monotherapy	
			NCT00036543	Phase III, randomized, double-blind, placebo-controlled, monotherapy	
			NCT00134056	Phase III, randomized, double-blind, placebo-controlled, plus docetaxel	
			NCT00554229	Phase III, randomized, double-blind, placebo-controlled, monotherapy	
			NCT00626548	Phase II, single-arm, monotherapy	
			NCT00617669		
			NCT00050297		
**Glioma**	Atrasentan	ETAR	NCT00017264	Phase I	[119]
**Glioblastoma**	Macitentan	ETAR and ETBR	NCT01499251	Phase I, open-label, single-arm, plus temozolomide	[120]
			NCT02254954	Phase I, open-label, single centre, plus temozolomide and radiotherapy	
**Biliary tract**	SPI-1620	ETR agonist	NCT01773785	Phase 2, multicentre, open-label, plus docetaxel	[121]
**Solid tumors**	SPI-1620	ETBR agonist	NCT00613691	Phase I, open-label, single-arm	

On the basis of interesting preclinical data [122], a clinical trial investigating the effect of irinotecan, fluorouracil and leucovorin (FOLFIRI) with or without zibotentan in metastatic colorectal cancer (Table 2) has already been completed, but the results are still pending. The reasons for the failure of the clinical trials to date have already been discussed in a report by Rosano and Bagnato [10]. As the clinical trials started in advanced stages of the disease and following previous therapies, the molecular mechanisms may already be altered compared to the preclinical situation, where treatment usually starts earlier in cancer development. It has also been speculated that pharmacologic ETAR blockade may shift the balance toward ETBR signaling in the TME, which in turn may promote angiogenesis and allow immune evasion; a lack of biomarkers to identify patients who may benefit from ETAR blockade has also been discussed. In addition, the effect of ETAR blockade in immunocompromised mice in preclinical studies may differ from ETAR blockade in humans, as immune cells play an important role in carcinogenesis. In addition, particular pitfalls have been reported in individual clinical trials, such as screening difficulties associated with incomplete staging [107,123] and the administration of higher total doses of chemotherapy to patients in the placebo treatment arm compared with patients receiving ETAR blockade [109]. Although the overall results of these clinical trials were disappointing, a small group of patients appeared to benefit from a selective ETAR antagonist. In particular, atrasentan was shown to reduce prostate cancer-induced bone remodelling [103]. In addition, a specific subgroup of prostate cancer patients in the SWOG S0421 trial with particularly high markers of bone turnover showed significantly improved OS with 13 months (atrasentan) versus 5 months (placebo) and a hazard ratio (HR) of 0.33. The observed improvement in survival in these patients may be due to the fact that highly elevated markers of bone turnover indicate a greater burden of bone metastases, which are a common cause of morbidity such as pain or fractures [124].

**Table 2:** Ongoing clinical trials targeting the endothelin system in cancer

Trial name	Compound	Targeting	Cancer type	NCT number	Status
**Gemcitabine, nab-paclitaxel, and bosentan for the treatment of unresectable pancreatic cancer**	Bosentan	ETAR and ETBR	Pancreatic	NCT04158635	Recruiting
**Study to evaluate the effect of bosentan on the pharmocokinetics of lurbinectidine in patients with advanced solid tumors**	Bosentan	ETAR and ETBR	Solid tumors	NCT05072106	Unknown
**ENB003 plus pembrolizumab phase 1b/2 in solid tumors**	ENB003	ETBR	Melanoma, ovarian- and pancreatic cancer	NCT04205227	Active, not recruiting
**Irinotecan hydrochloride, flourouracil, and leucovorin calcium with or without zibotentan in treating patients with metastastatic colorectal cancer (FOLFERA)**	Zibotentan	ETAR	Colorectal cancer	NCT01205711	Completed, no results posted

In another study in which atrasentan was administered in combination with interferon (IFN)-α for the treatment of renal cell carcinoma (RCC), exploratory analysis showed that median OS worsened dramatically by 20 months in patients with increasing VEGF levels in combination with stagnant ET1 levels, compared with patients with decreasing VEGF levels (2.2 months versus 22.2 months) [108]. Since ETAR receptor activation induces VEGF expression via HIF-1α [125] and VEGF drives angiogenesis [126], one could speculate that elevated VEGF levels in this particular patient subset enabled tumor vascularization to rapidly drive cancer growth. Selective blockade of ETAR may have led to compensatory up-regulation of ETBR, which is involved in the secretion of ET1. However, why VEGF levels increased and ET1 levels remained low in this small group of patients remains an open question. As IFN-α is no longer standard of care in the treatment of renal cell carcinoma, further investigation is required and a different approach needs to be taken. Two phase two clinical trials testing the ETAR antagonists atrasentan and zibotentan with Pegylated liposomal doxorubicin (PLD) and carboplatin or paclitaxel as second-line therapy for advanced and refractory ovarian cancer failed to demonstrate a clear benefit for patients treated with an ETAR antagonist [109,115]. However, in the smaller atrasentan trial, three out of a total of 26 patients showed an objective response and in the larger zibotentan trial, four out of a total of fifty-nine patients showed a complete remission compared to none out of 61 patients who received placebo. Although these patient numbers appear very small, they indicate a subgroup of ovarian cancer patients who may benefit from ETAR blockade in addition to conventional chemotherapy. Further biomarker studies are required to identify these patients. In a study of ET1 gene expression in 201 tumor samples of non-small cell lung cancer (NSCLC), overexpression of the endothelin axis compared to normal lung tissue was found to be a negative prognostic factor for both OS (*P*=0.03) and disease-free interval (*P*=0.04) [127].

In another study, the precursor molecule big ET1 was detected in the plasma of 30 NSCLC patients and it was found that patients with elevated levels of big ET1 had a worse outcome than patients with lower levels of big ET1 [128]. These intriguing preclinical data prompted the initiation of two clinical trials in advanced NSCLC: a single-arm phase I/II clinical trial evaluating the combination of carboplatin and paclitaxel in first-line with atrasentan, and a placebo-controlled phase II trial in which patients received second-line pemetrexed either with a placebo or in combination with zibotentan. Although both studies showed an acceptable safety profile, the clinical trials failed to demonstrate a clinical effect of the ETAR antagonists [129]. In a more recent study using xenograft mice, EGFR-mutated lung cancer cells were observed to produce ET1 when treated with EGFR inhibitors. The researchers proposed the ET axis as a mechanism of drug resistance in EGFR-mutated NSCLC through vasoconstriction, reducing drug delivery to the tumor. Specifically, the addition of bosentan to gefitinib, an EGFR inhibitor, was found to significantly improve tumor perfusion and drug delivery, in addition to successfully reducing tumor size and preventing tumor recurrence compared to treatment with gefitinib alone [106,110]. These promising preclinical data have yet to lead to a clinical trial in EGFR-mutated, drug-resistant NSCLC, which could potentially yield interesting results.

## ETBR antagonists

Clinical studies targeting ETBR are rather scarce, and the results are limited to a few studies on melanoma (Table 1). The first study was a proof-of-concept study in which five patients received intralesional injections of BQ788, a selective ETBR antagonist, in increasing doses into melanoma skin metastases compared with injections with phosphate buffered saline (PBS) into different skin lesions of the same patient [111]. The lesions were then surgically removed at different time points. The only patient who experienced a reduction in lesion size was the only one who was treated for 14 days instead of 7 days. In addition, an increase in blood vessel and lymphocyte infiltration was noted in some patients. This small, translational study could provide an indication of a possible anti-tumor effect of ETBR blockade in melanoma. Further studies with more patients and longer time to resection should provide interesting results. The second study is a Phase I trial of the antibody-drug conjugate (ADC) DEDN6526A in patients with metastatic or unresectable cutaneous, mucosal or uveal melanoma [130]. This ADC consists of an anti-ETBR antibody conjugated to monomethyl auristatin E (MMAE), an anti-mitotic agent. Of 44 patients, 6 patients (13%) showed radiographic partial remission (PR), and of 24 patients who had positron emission tomography (PET) available, 16 patients (62%) showed partial metabolic response (PMR). The patients who responded to ADC had high ETBR expression, but ETBR expression alone does not appear to predict response to therapy, as many patients who did not respond also had elevated ETBR expression. As this study did not show an overall response rate (ORR) different from standard chemotherapy, this particular ADC was not developed further. However, it was discussed that targeting ETBR through other modalities such as chimeric antigen receptor T cells (CAR-T) may be an option for future investigation. An ongoing phase 1b/2 clinical trial is investigating the effect of ENB003, an ETBR antagonist, in combination with pembrolizumab, an ICI, in solid tumors (Table 2).

Although other cancers are also approved, this study focuses on metastatic melanoma, platinum-resistant ovarian cancer and pancreatic cancer, cancers in which ETBR is either highly expressed [101,131] or known to correlate with poorer survival [85]. Buchanovich et al. demonstrated the correlation between ETBR expression and poor OS in ovarian cancer, but also showed a correlation with tumor infiltrating lymphocyte (TIL) deficiency, which was reversed by ETBR blockade [85], which improved improving survival in mice. Given the indispensable role of T cells in the mode of action of ICI, the results of this clinical trial, which is expected to be completed in 2026, are eagerly awaited.

## Dual ET receptor antagonists

Since ETAR is highly expressed on many types of cancer cells and ETBR is expressed in the TME, the exploration of dual receptor antagonists for the treatment of cancer seems tempting. One advantage of this approach is the large clinical experience with dual receptor antagonists such as bosentan and macitentan, which have revolutionized the treatment of pulmonary hypertension and are frequently used clinically [132,133]. However, similar to the blockade of a single ET receptor, positive results from clinical trials on dual ET blockade are still pending. Results from two clinical trials in metastatic melanoma are available [112,113]. After a phase II study in which bosentan was investigated as a monotherapy showed modest results [112], bosentan was investigated in combination with dacarbazine, a chemotherapeutic agent, in a randomized, double-blind, placebo-controlled clinical trial [113]. Disappointingly, the time to progression (TTP) in the bosentan group was only 1.6 months, while the TTP in the cohort receiving placebo was 2.8 months. In addition, no analysis of ET receptor status or other investigation of a potential biomarker was performed. Following promising preclinical studies in which an impressive 46 out of a total of 48 (96%) glioblastoma-bearing mice showed no signs of residual disease after treatment with macitentan and temozolomide [134], a phase I clinical trial showed an acceptable safety profile, an ORR of 13.9% and an OS of 9.4 months [120]. Fully aware of the pitfalls of comparing data from different clinical trials, a pooled analysis showed significantly lower ORR (4-7%) and OS rates (5–7 months) from other clinical trials [135], and not enough patients were included to draw meaningful conclusions on efficacy. Another clinical trial investigating macitentan, radiotherapy and temozolomide in glioblastoma patients was terminated prematurely due to insufficient enrollment (Table 1). Further clinical trials with larger numbers of patients could provide more information on whether dual ET blockade can improve the treatment of glioblastoma. An ongoing phase I trial is investigating the effect of bosentan in combination with gemcitabine and albumin-bound (nab)-paclitaxel in unresectable pancreatic cancer (Table 2). Since both ETAR and ETBR are [101] expressed in pancreatic cancer, an improvement in ORR may be observed. Interestingly, all studies with dual ET receptor antagonists used much higher doses than those approved for pulmonary hypertension. Although the doses tested were up to 30 times higher than those used for the treatment of pulmonary hypertension, the number of adverse events (AEs) did not exceed the expected threshold and were mostly related to the disease itself or the concomitant agent in the study.

## Further approaches

A contrasting approach to ET in cancer research was taken by Kim et al. In this phase II study, SPI-1620, an ETBR agonist, was investigated in addition to docetaxel in bile duct cancer [121]. The addition of an ETBR agonist to the treatment was an attempt to improve drug penetration and distribution in the tumor, as failure to achieve adequate cytotoxic concentrations in the tumor has been identified as a potential mechanism mediating treatment failure in bile duct cancer. However, the preclinical data on the improvement of tumor perfusion by ETBR are unclear: the EBTR agonist IRL-1620 improves tumor perfusion in some animal models of [136,137] and causes severe vasoconstriction in other [138,139], whereas bosentan, a dual ETR antagonist, significantly improved tumor perfusion in pancreatic cancer [77]. In addition to a high incidence of grade 3 or 4 adverse events, the trial was terminated prematurely because the pre-specified goal of a median progression-free survival of 5 months could not be achieved. Other, less sophisticated concepts for targeting the ET system in cancer research such as ECE-1 inhibition by specific inhibitors [140–142] or polyphenols from natural products such as green tea [143] or up-regulation of ET clearance mechanisms by NEP have not yet triggered a clinical trial [144–146].

## Prospects and limitations

Preclinical studies can be optimized by using genetically engineered mouse models or humanized xenograft mouse models instead of immunocompromised mouse models. As previously discussed, immunocompromised xenograft models may not conclusively represent the human TME given the central role of ET in the immune response to cancer. In addition, particularly in the context of lung cancer research, precision-cut lung slices (PCLS) are an emerging concept to study the effect of antitumor agents in human *ex vivo* experimental models. Clinical trials should be carefully designed, ideally in a translational setting, to identify potential biomarkers in order to select patients likely to benefit from ET receptor antagonism. Specific gene signatures [64], a favorable immune cell landscape in the TME [75,94,98], specific ETBR isoforms [147] or molecular markers such as mir-30a [61] are potential targets for future translational studies. In addition, innovative theranostic approaches could improve patient prognosis by using radioligands and PET imaging to stratify patients eligible for ET receptor antagonism. Hautiere et al. developed a novel radioligand targeting ETAR and demonstrated its efficacy in μPET-(positron emission tomography) CT in an orthotopic glioblastoma mouse model. In addition, Vivier et al. showed a similar efficiency of their newly developed PET/near-infrared fluorescence probe targeting both ETAR and ETBR in mice with ETAR and ETBR overexpressing Chinese hamster ovary (CHO) cells [148,149]. A future clinical trial investigating radioligands to detect ET receptor expression as a means of stratifying patients could help identify patients who could potentially benefit from ET receptor antagonism and should ideally be designed in a longitudinal design and embedded in a clinical trial investigating ET receptor antagonists. As the preclinical data suggest, ET receptor antagonists should ideally be used in combination with other established and novel treatments such as conventional chemotherapy, ICIs or PARP inhibitors. In particular, the homing of T cells into the TME as a result of the ET receptor antagonist [85] calls for the combination with ICIs in a clinical trial. The early phase clinical trial investigating the ETBR antagonist ENB003 in combination with pembrolizumab in certain solid tumors could be just the beginning (Table 2). In addition, dual ET receptor antagonism should be considered in clinical trials of solid cancers expressing both ETAR and ETBR. Since ETAR is mainly expressed on cancer cells and ETBR in the TME, blocking both receptors could have synergistic effects and attack the cancer from multiple sides. One possible explanation for the discrepancy between preclinical and clinical study results could be the different tumor stage used for data collection. In general, the preclinical data comes from tumors treated at an early stage, as opposed to the clinical trials, which mostly involve patients at more advanced stages. Considering this potential factor contributing to the discrepancy, it seems worthwhile to investigate a trial in an adjuvant setting, although ET antagonists have not yet proven their efficacy in metastatic disease.

Previous research from our laboratory has shown in various mouse models that lung cancer is often associated with vascular remodeling and pulmonary hypertension (PH) due to the release of chemokines caused by cross-talk between cancer cells and immune cells. In addition, 250 of 512 patients showed enlargement of the pulmonary artery, an indirect sign of PH [150]. Recent studies have shown that lung cancer patients with signs of PH have worse OS [151]. Given the new concept of lung cancer-associated PH, clinical trials targeting the endothelin axis in these patients seem tantalizing, but as right heart catheterization remains the gold standard for PH diagnosis, a clinical trial in this patient population remains difficult.

## Conclusion

In summary, the ET system plays a critical role in every facet of the TME, influencing cellular responses to hypoxia, EMT regulation, and the overall cellular composition. Research emphasizes the importance of the ET axis in cancer, with promising results from animal models leading to clinical trials for various solid cancers. However, the consistently disappointing results of these trials, in contrast to preclinical studies, underscore the complicated nature of the endothelial system in cancer. Despite more than two decades of extensive research, a knowledge gap remains, underscoring the need for additional preclinical and clinical studies to gain clarity on this complex aspect of cancer biology.

## Data Availability

This is a review article.

## Abbreviations

α-SMA: α-smooth muscle actin
ADAM 17: a disintegrin and metalloproteinase 17
ADC: antibody-drug conjugate
AE: adverse event
CAF: cancer-associated fibrocyte
CAR-T: chimeric antigen receptor T cells
COX: cyclooxygenase
DC: dendritic cell
EC: endothelial cell
ECE: endothelin-converting enzyme
ECM: extracellular matrix
EGFR: epidermal growth factor receptor
EMT: epithelial-to-mesenchymal transition
ET: endothelin
ETAR: endothelin A receptor
ETBR: endothelin B receptor
FAK: focal adhesion kinase
FC: fibrocyte
FOLFIRI: irinotecan, fluorouracil, leucovorin
FOXO: forkhead box O
GSK: glycogen synthase kinase
HG-SOC: high-grade serous ovarian carcinoma
HIF-1α: hypoxia-inducible factor-1alpha
HR: hazard ratio
HRE: hypoxia response element
HUVEC: human umbilical vascular endothelial cell
ICAM1: intercellular adhesion molecule1
ICI: immune checkpoint inhibitor
IFN: interferone
IL: interleukin
ILK: integrin-linked kinase
IP3: inositol 1,4,5-triphosphate
IQGAP: IQ-domain GTPase-activating protein
MAPK: mitogen-activated protein kinase
MiRNA: microRNA
MMAE: monomethyl auristatin E
MMP: matrix metalloproteinase
mTOR: mammalian target of rapamycin
NCT: clinicaltrials.gov identifier
NEP: neutral endopeptidase
NF-κB: nuclear factor ‘kappa-light-chain-enhancer’ of activated B-cell
NO: nitric oxide
NSCLC: non-small cellular lung cancer
ORR: overall response rate
OS: overall survival
PARP: poly-ADP-ribose polymerase
PBS: phosphate buffered saline
PCLS: precision-cut lung slices
PD-1: programmed death-1
PDL1: programmed death ligand -1
PET: positron emission tomography
PG: prostaglandin
PH: pulmonary hypertension
PHD: prolyl hydroxylase domain
PI3K: phosphatidylinositol 3-kinase
PLC: phospholipase C
PLD: pegylated liposomal dacarbazin
PMR: partial metabolic response
PR: partial remission
RCC: renal cell carcinoma
STAT3: signal transducer and activator of transcription 3
TAM: tumor associated macrophage
TIL: tumor-infiltrating lymphocyte
TIMP: tissue inhibitor of metalloproteinase
TME: tumor microenvironment
TTP: time to progression
uPA: urokinase plasminogen activator
VEGFR: vascular epithelial growth factor receptor
YAP: yes-associated protein
ZEB: zinc finger E-box-binding homeobox
